# On Modern Progress in Ophthalmic Medicine and Surgery

**Published:** 1894-06-16

**Authors:** Robert Brudenell Carter

**Affiliations:** Consulting Ophthalmic Surgeon to St. George's Hospital


### On Modern Progress in Ophthalmic Medicine and Surgery.

By Robert Brudenell Carter, F.R.C.S., Consulting Ophthalmic Surgeon to St. George's Hospital.

The excision of a portion of the iris, as described in the last article, is followed by a variable amount of bleeding into the anterior chamber, an amount sometimes scarcely perceptible, sometimes sufficient to occupy the pupillary space and to conceal the surface of the cataract. In the latter case, the surgeon must promote the escape of the blood by gentle pressure upon the posterior lip of the external wound, pressure which may be conveniently exercised by the spoon of the curette, and the surface of the cornea may also be subjected to gentle pressure or stroking with the back of a tortoiseshell spoon, while the issuing blood is taken up from the surface by a morsel of sponge. As soon as the margin of the pupil can be seen, the surgeon introduces a cystitome, with its point directed laterally, into the anterior chamber, and carries it well underneath the lower pupillary margin before turning its sharp point against the anterior capsule, which it is then made to divide from below upwards, nearly or quite to the equator. As this is done, the released lens tissue is generally seen to bulge forward and approach the incision; but it is nevertheless desirable, in most cases, to carry the point of the cystitome through the capsule, in the equatorial region of the lens, along a line about equal in length to the external incision. The cystitome should then be turned sideways, and cautiously withdrawn from the eye in such a manner that its point cannot become engaged in any of the tissues.

The next step in the operation is that which makes the largest demand upon the skill and muscular sense of the surgeon. The object in view is to extrude the lens by gentle pressure, exerted in such a manner that there may be no escape of vitreous, and that the lens may continuously advance while the pressure is being applied. This is best done by the back of the tortoiseshell spoon already mentioned, a spoon which has a slight amount of elastic flexibility, and which is set in a firm ivory handle. Taking this in the right hand, the surgeon raises the upper lid with the tips of two fingers of the left hand, and permits the tip of the left index finger to rest upon the eyeball immediately behind the incision. The back of the spoon is then applied to the surface of the cornea about half way between its centre and its lower margin, and the pressure is first made directly backwards, so as to induce the lens to turn as if upon a horizontal axis, and to present its upper margin at the incision. The slightest counter-pressure with the tip of the left index causes the wound to gape, and, by the combination of the two procedures, the lens fairly engages itself between the lips of the wound. As soon as it does this the direction of the spoon pressure may be changed, and the spoon so used as gently to stroke the corneal surface from below upwards, and to follow the lens as it escapes. As soon as the escape is imminent, that is to say, as soon as the diameter of the lens has passed through the incision, the pressure may be relaxed, and the final extrusion will be accomplished by the elasticity of the tissues concerned. An experienced operator will be able to use pressure with much greater freedom than one who has still his experience to gain, the educated muscular sense of the former telling him precisely how much it will be safe to do, and at what point the vitreous would be exposed to danger. It follows that an inexperienced operator should never err in the direction of making too small an incision, because a comparatively large one greatly facilitates the escape of the cataract, and therefore diminishes the amount of pressure likely to be required.

As the lens passes out of the eye the surgeon receives it in the tortoiseshell spoon, and lays it aside. In some cases, the lips of the wound will immediately fall into perfect apposition, in others they will be slightly separated by a tendency to hernia of the vitreous, still contained within its hyaloid membrane. In the latter case the lids must at once be closed, and gently supported for a few seconds, until the vitreous has had time to return to its natural position. The operator then raises the lid and inspects the pupillary space, which may either be perfectly free, or may contain a portion of the cortical substance of the lens, with or without some admixture of blood from the iris. In the first case a certain amount of vision would be at once restored, insomuch that the patient should be able to see the fingers of the surgeon, held at about a foot from the eye, to count them, to distinguish a thumb from a little finger, and to see a ring upon either. If this, or something like this cannot be done, the pupillary space is still occupied by matter which it is desirable to remove. Very gentle rotatory finger friction should first be applied to the cornea through the closed lids, and then the tortoiseshell spoon may be once more employed to press out what remains, its action being assisted by the fine silver spoon of the curette introduced just within the anterior chamber. If possible, procedures of this kind should be continued until the pupil is clear, and the above-mentioned visual tests have been responded to satisfactorily; but any appearance of vitreous in the incision, and still more any loss of it, should call for immediate closure of the lids, even at the cost of leaving something behind. Residual cortex may set up a certain amount of iritis, but this, if antiseptic precautions have been duly observed, is scarcely likely to be more than a temporary inconvenience.

The methods of external dressing employed by different surgeons differ greatly, and I shall not attempt more than to give a description of the method which I employ myself, and at which I have arrived after many trials of different plans. The lids being closed, I place over their line of junction and upon the eyelashes a strip of fine cambric from an old handkerchief, about an inch long and a quarter of an inch broad, freely smeared with sanitas vaseline. Over this I place a small horizontal roll of cotton wool as long as the cambric and as thick as an ordinary cedar pencil. This is kept in place by three or four strips of Johnson's Gelatole plaster, each about a third of an inch wide and three or four inches long, the first vertical, crossing the centre of the palpebral fissure from forehead to cheek, the second and third oblique, crossing each other saltire-wise; and the fourth, if applied, horizontal. Unless there be some symptom to call for interference, I do not remove this dressing for forty-eight hours, when it can be taken off in a minute by soaking the plasters with warm water. The greased slip of cambric does not adhere to the lid edges or to the eyelashes, and the wound, generally speaking, is found to be healed. When this is so, I feel justified in saying to the patient that vision is secure.

